# Expression of hepatocytic- and biliary-specific transcription factors in regenerating bile ducts during hepatocyte-to-biliary epithelial cell transdifferentiation

**DOI:** 10.1186/1476-5926-9-9

**Published:** 2010-12-02

**Authors:** Pallavi B Limaye, William C Bowen, Anne Orr, Udayan M Apte, George K Michalopoulos

**Affiliations:** 1 Department of Pathology, School of Medicine, University of Pittsburgh School of Medicine, Pittsburgh, PA 15261, USA; 2 Department of Pharmacology, Toxicology, and Therapeutics, University of Kansas Medical Center, Kansas City, KS 66160, USA

## Abstract

**Background:**

Under compromised biliary regeneration, transdifferentiation of hepatocytes into biliary epithelial cells (BEC) has been previously observed in rats, upon exposure to BEC-specific toxicant methylene dianiline (DAPM) followed by bile duct ligation (BDL), and in patients with chronic biliary liver disease. However, mechanisms promoting such transdifferentiation are not fully understood. In the present study, acquisition of biliary specific transcription factors by hepatocytes leading to reprogramming of BEC-specific cellular profile was investigated as a potential mechanism of transdifferentiation in two different models of compromised biliary regeneration in rats.

**Results:**

In addition to previously examined DAPM + BDL model, an experimental model resembling chronic biliary damage was established by repeated administration of DAPM. Hepatocyte to BEC transdifferentiation was tracked using dipetidyl dipeptidase IV (DDPIV) chimeric rats that normally carry DPPIV only in hepatocytes. Following DAPM treatment, ~20% BEC population turned DPPIV-positive, indicating that they are derived from DPPIV-positive hepatocytes. New ductules emerging after DAPM + BDL and repeated DAPM exposure expressed hepatocyte-associated transcription factor hepatocyte nuclear factor (HNF) 4α and biliary specific transcription factor HNF1β. In addition, periportal hepatocytes expressed biliary marker CK19 suggesting periportal hepatocytes as a potential source of transdifferentiating cells. Although TGFβ1 was induced, there was no considerable reduction in periportal HNF6 expression, as observed during embryonic biliary development.

**Conclusions:**

Taken together, these findings indicate that gradual loss of HNF4α and acquisition of HNF1β by hepatocytes, as well as increase in TGFβ1 expression in periportal region, appear to be the underlying mechanisms of hepatocyte-to-BEC transdifferentiation.

## Background

Transdifferentiation of the liver epithelial cells (hepatocytes and biliary cells) into each other provides a rescue mechanism in liver disease under the situations where either cell compartment fails to regenerate by itself. We have previously reported transdifferentiation of hepatocytes into biliary epithelial cells (BEC) both in *in vivo *rat model using biliary toxicant 4,4'-methylenedianiline [diaminodiphenyl methane, (DAPM)] followed by biliary obstruction induced by bile duct ligation (BDL) [1] and *in vitro *using hepatocyte organoid cultures treated with hepatocyte growth factor (HGF) and epidermal growth factor (EGF) [2-4]. Other investigators have also demonstrated hepatocyte-to-BEC transdifferentiation in hepatocyte cultures [5] and following hepatocyte transplantation in spleen [6]. In humans, chronic biliary liver diseases (CBLD) characterized by progressive biliary epithelial degeneration are also known to be associated with formation of intermediate hepatobiliary cells expressing both hepatocytic and biliary specific markers [7-9]. However, the mechanisms promoting such hepatocyte to BEC transdifferentiation (or vice versa) are not completely understood. In the current study, by repeatedly injuring biliary cells by minimally toxic dose of DAPM administered to rats we established a novel rodent model resembling CBLD [10]. DAPM selectively injures biliary cells because toxic metabolites of DAPM are excreted in bile [10,11].

Orchestrated network of liver-enriched transcription factors is known to play an important role in pre- and postnatal liver development as well as in lineage specification of hepatoblasts into hepatocytes and BECs [12,13]. Studies with knockout mice have shown that hepatocyte nuclear factor (HNF) 1α and HNF4α regulate transcription of genes essential for the hepatocytic lineage [14-16] whereas HNF1β and HNF6 are involved in development of the gallbladder and bile ducts [17-19]. In the present study, the expression of hepatocyte- and biliary-specific HNFs is examined during reprogramming of cell lineage during transdifferentiation using DAPM + BDL and repeated DAPM treatment models.

Gradient of TGFβ expression regulated by Onecut transcription factor HNF6 in ductal plate hepatoblasts during embryonic liver development is crucial for biliary differentiation [20]. In the present study, TGFβ1 and HNF6 expression pattern was studied in order to determine if similar mechanism is recapitulated during hepatocyte to BEC transdifferentiation in the adult liver. The likely source of hepatocytes capable of functioning as progenitor cells in the event of compromised biliary regeneration is investigated by assessing expression of biliary specific keratin CK19.

To examine if hepatocytes transdifferentiate into biliary epithelium after repeated administration of DAPM, dipeptidyl peptidase IV (DPPIV) chimeric rats were utilized that normally carry DPPIV-positive population of only hepatocytes derived from donor DPPIVpositive rats [21,1-3]. Neither the hepatocytes nor the BECs express DPPIV in the recipient DPPIV negative rats. Thus, appearance of biliary epithelial cell clusters positive for the hepatocyte marker DPPIV provides strong evidence that BEC are derived from hepatocytes.

## Results

### Histological and functional bile duct damage after DAPM administration

**Figure 1:**
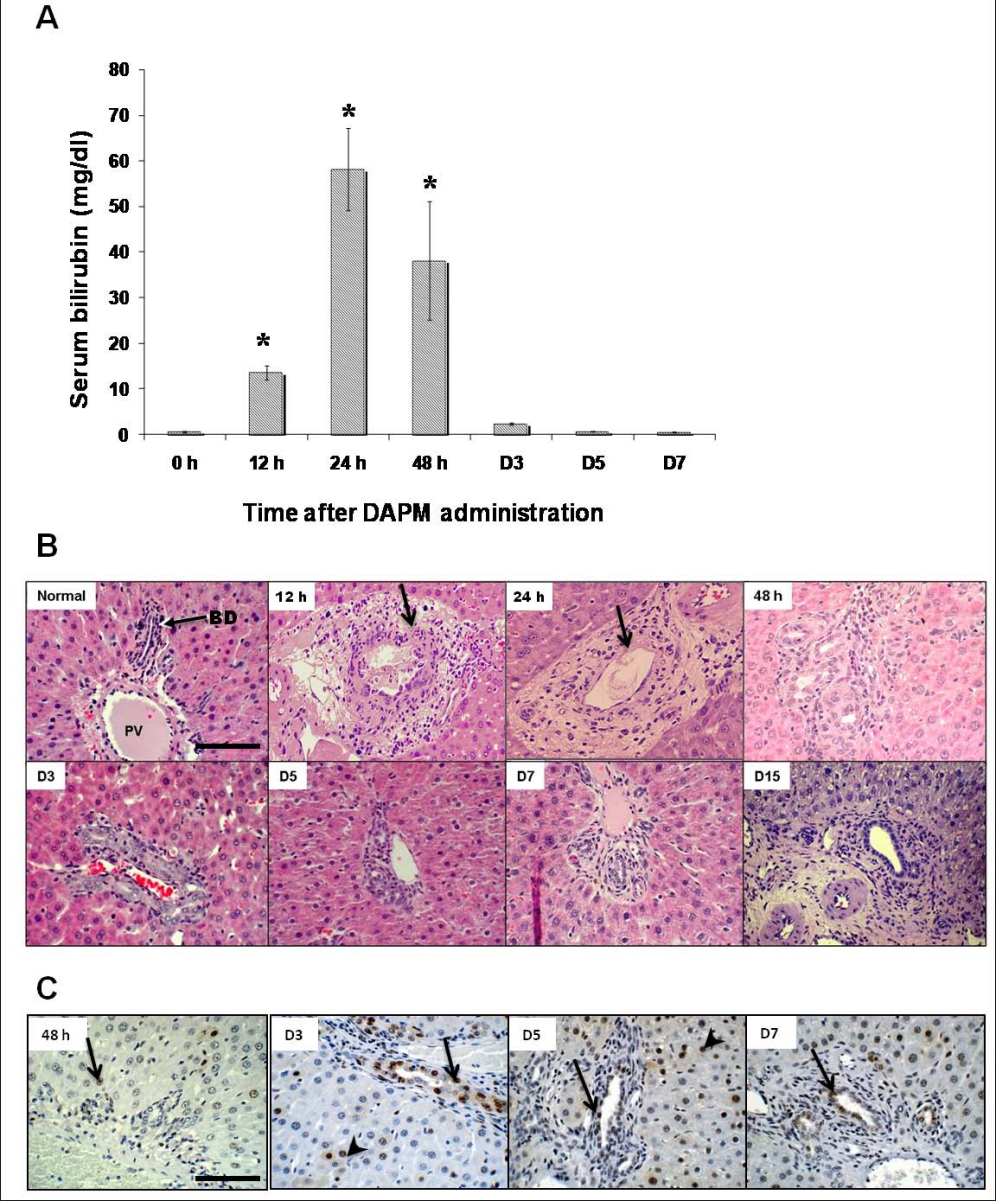
**Biliary injury and regeneration following DAPM toxicity**. **(A)** Serum bilirubin levels indicative of biliary injury after DAPM (50 mg/kg) administration in F344 rats over a time course. * indicates statistical difference from the 0h control (P ≤ 0.05). **(B) **Histopathology of the liver following DAPM toxicity (50 mg/kg) depicted by H&E staining. Arrow points to the biliary injury. **(C) **Biliary regeneration after DAPM (50 mg/kg) toxicity depicted by PCNA immunohistochemistry. Brown staining indicates PCNA positive cells. Thin arrow indicates regenerating biliary ductules. Arrowhead points to the hepatocyte proliferation. Scale bar = 100 μm.

Biliary toxicity induced by single administration of DAPM (50 mg/kg, ip) was monitored by elevations of serum bilirubin and histopathological observations over a time course. Maximum biliary injury in terms of serum bilirubin was apparent by 24 h and consistently stayed high till 48 h after DAPM (Figure 1A). By day 7, rats appeared to recover from toxicity as indicated by regressing serum bilirubin levels (Figure 1A). Histopathological observations revealed biliary cell necrosis as early as 12 h after DAPM. Necrosis was accompanied by ductular swelling and inflammation. Some damage to the hepatocytes was also observed in the form of bile infarcts. However, the serum ALT elevations were minimal suggesting hepatocyte injury by DAPM was secondary (Additional File 1, Figure S1). Based on the quantitative analysis, 70% bile ducts were injured by DAPM at 24 h after DAPM. At 48 h, the bile ducts appeared to be repairing from injury (Figure 1B). The PCNA analysis indicated that the biliary cells begin cell division at 48 h and continue till day 7 (Figure 1C). Based on these findings, we chose to administer DAPM (50mg/kg, ip) every 2 days for total 3 times in order to inflict repeated biliary injury and simultaneously impairing their ability to regenerate themselves. It should be noted that it is the same dose of DAPM that was used in our previous study using DAMP + BDL injury model [1].

### Appearance of DPPIV-positive bile ducts after repeated administration of DAPM

**Figure 2:**
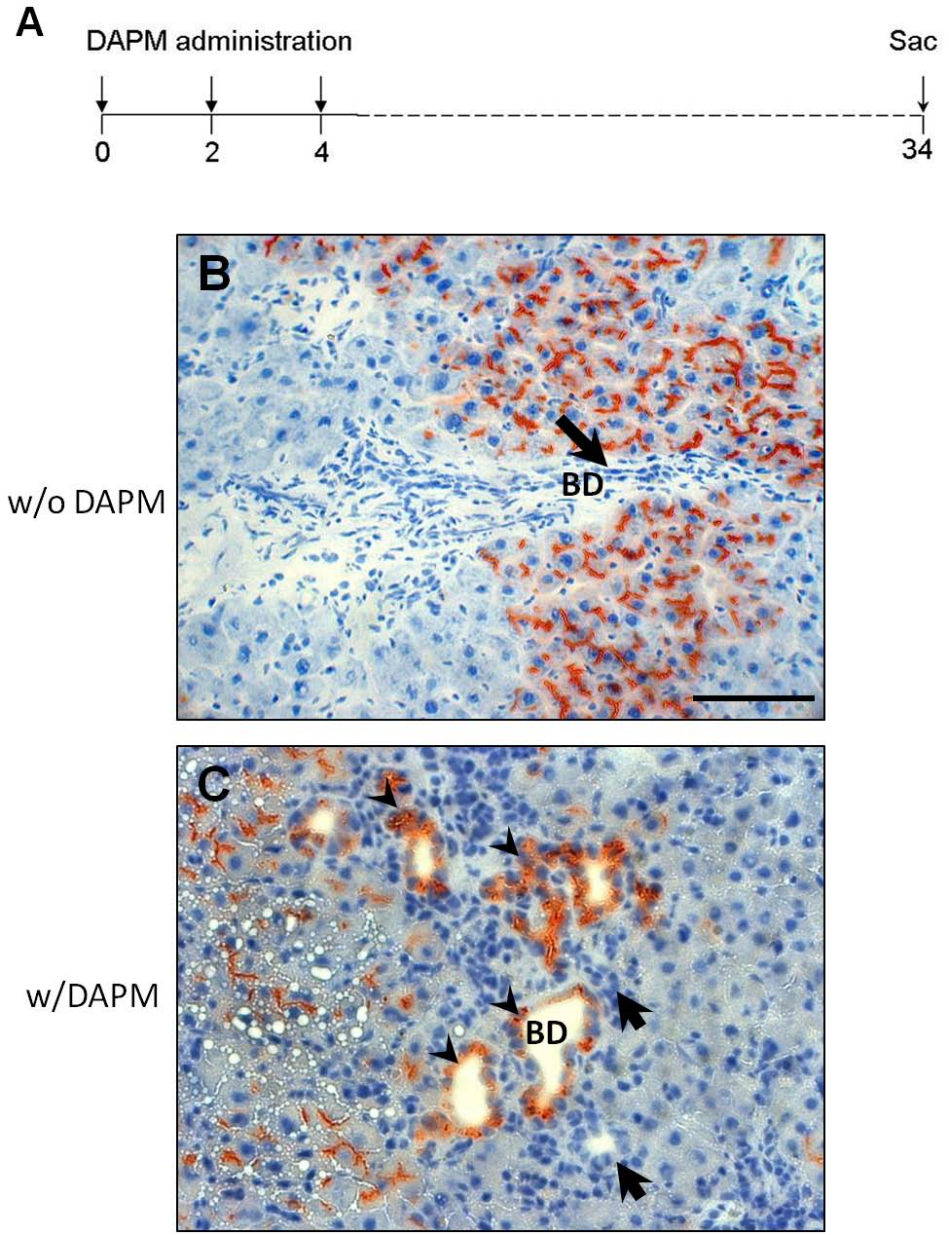
**Appearance of DPPIV in bile ducts cells after repeated DAPM administration (DAPM × 3)**.**(A)** Schematic representation of repeated DAPM administration protocol. DAPM (50 mg/kg) administered at day 0, 2, and 4 to the DPPIV chimeric rats. Rats sacrificed at day 30 after the last DAPM injection. DPPIV staining before **(B)** and after **(C)** repeated DAPM administration to the DPPIV chimeric rats. Arrowheads point to the DPPIV positive bile ducts. Arrows indicate DPPIV negative bile ducts. The number of DPPIV positive bile ducts was determined after counting DPPIV positive bile ductules in liver sections obtained from different lobes of liver from 3 individual rats separately. None of the bile duct cells of the DPPIV chimeric rats were positive before DAPM treatment. ~20% bile ducts were noted to be DPPIV positive after DAPM × 3 protocol. Scale bar = 100 μm.

The DPPIV chimeric rats were injected with DAPM at day 0, day 2, and day 4 (Figure 2A). On day 30 after the last injection of DAPM the rats were sacrificed and the liver sections from various lobes were examined for DPPIV positivity. Before DAPM administration, there was 40%-50% engraftment of the DPPIV-positive hepatocytes as reported before and none of the biliary cells were DPPIV-positive (Figure 2B). After DAPM repeated administration ~20% of the bile ducts turned DPPIV-positive indicating that they are derived from DPPIV positive hepatocytes (Figure 2C).

### Periportal hepatocyte expression of CK19

**Figure 3:**
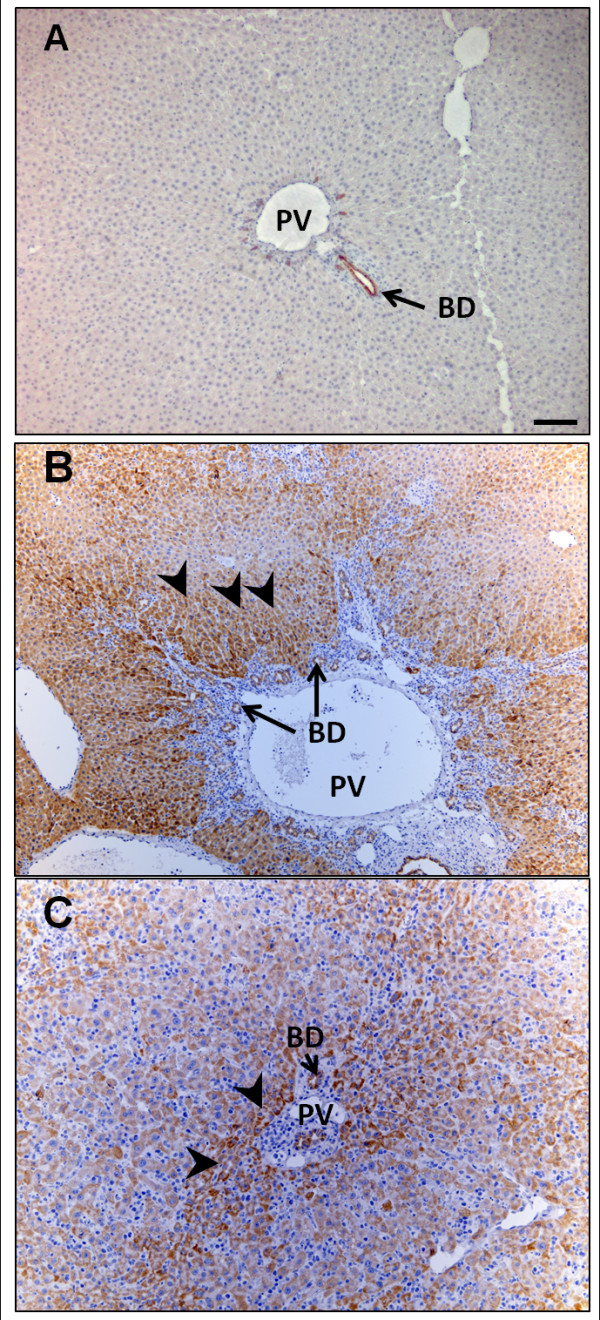
**Localization of CK19 following DAPM + BDL or repeated DAPM treatment (DAPM × 3)**. **(A) **Normal rat liver (NRL), (**B**) liver from DAPM + BDL treated rat, **(C) **liver from repeated DAPM treatment (DAPM x3). Brown color indicates CK19 positive staining. **Arrows **indicate bile duct staining. Arrowheads indicate hepatocytic staining. PV, portal vein; BD, bile duct. Scale bar = 100 μm.

CK19 was expressed only in BEC in the normal liver (Figure 3A). However, after DAPM treatment protocol, selective periportal hepatocytes were also strongly positive for CK19 in addition to the BEC (Figure 3B and 3C). Periportal hepatocytic CK19 staining was not uniform across the liver lobule. These findings indicate that the periportal hepatocytes only in the proximity of the affected biliary cells offer a pool of facultative stem cells capable of transdifferentiation to biliary cells.

### Hepatocyte-associated transcription factor HNF4 α expression in newly formed biliary ductules

**Figure 4:**
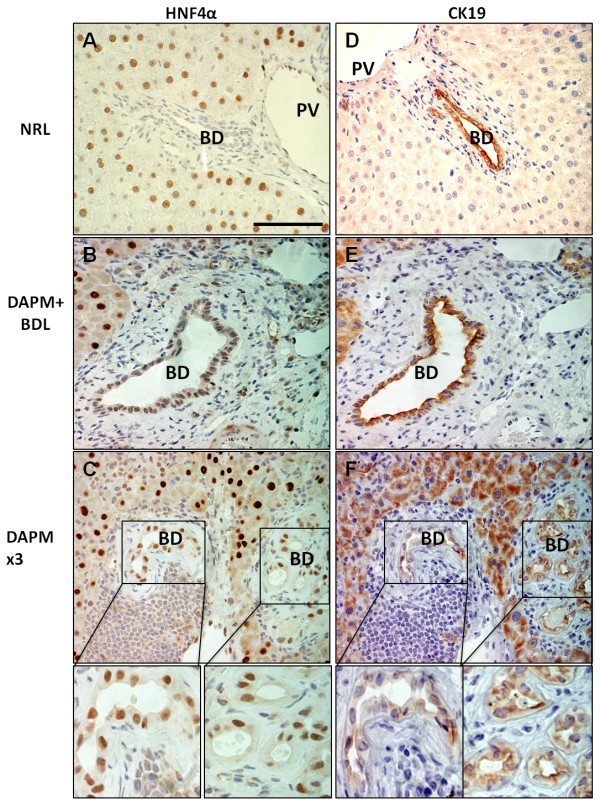
**HNF4α and CK19 immunohistochemistry**. Liver sections obtained from normal control rats (NRL, normal rat liver) **(A and D)**, rats that underwent DAPM + BDL treatment **(B and E)**, or repeated DAPM treatment (DAPM × 3) **(C and F)**. **B**, **E** and **C**, **F** are serial sections. Brown nuclear staining indicated HNF4α positive cells in the left panel. Brown cytoplasmic staining in the right panel indicates CK19 positive cells. NRL bile ducts are HNF4α- negative and CK19 positive. However, after DAPM + BDL and DAPM × 3 treatment bile ducts turn HNF4α positive along with CK19. In addition, periportal hepatocytes also turn positive for CK19 after BDL + DAPM and DAPM × 3 treatment. PV, portal vein; BD, bile duct. Scale bar = 100 μm.

Figure 4 depicts the HNF4α (Figure 4A, B, and 4C) and CK19 (Figure 4D, E, and 4F) stainings on the serial liver sections. In the normal rat liver, nuclear HNF4α expression is observed only in the hepatocytes (Figure 4A). However, the biliary ductules undergoing repair after repeated DAPM administration or DAPM + BDL show incorporation of cells resembling hepatocyte morphology that also had HNF4α positive staining (Figure 4B and 4C, respectively). In Figure 4C and 4F there is a panel of ductules in which only some of the cells in a duct are HNF4α positive and only some of the cells are CK19 positive (with overlap between some of the cells).

### Appearance of biliary-specific transcription factor HNF1β in hepatocytes intercalated within biliary ductules

**Figure 5:**
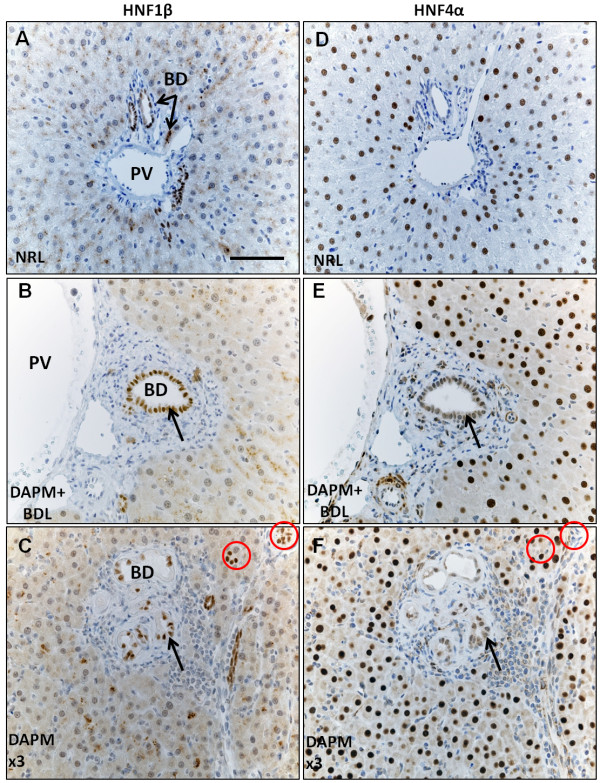
**HNF1β and HNF4α immunohistochemistry on serial liver sections**. **(A) **normal control rats (NRL, normal rat liver), **(B) **rats that underwent DAPM + BDL treatment, or **(C) **repeated DAPM treatment (DAPM × 3). HNF1β and HNF4α coexpressing cells are pointed by an arrow. HNF1β positive but HNF4α negative bile ducts pointed by circles. PV, portal vein; BD, bile duct. Scale bar = 100 μm.

HNF1β staining is observed only in the biliary nuclei of the normal rat liver (Figure 5A) but not in the hepatocytes. After DAPM + BDL injury (Figure 5B) and repeated DAPM toxicity (Figure 5C), many cells which morphologically appear as hepatocytes are seen intercalated within biliary ductules that coexpress HNF4α, indicating their hepatocytic origin. Many (but not all) of these cells stain positive for HNF1β (Figure 5B and 5C). Notice the ductules marked with a thin arrow shown as an example have HNF1β stain, but are HNF4α- negative (Figure 5C and 5D). The cells coexpressing HNF1β and HNF4α appear bigger compared to the normal liver biliary cells, a characteristic of ductular reaction.

### Transforming growth factor beta 1 (TGFβ1) induction in the periductular region with no change in HNF6 staining

**Figure 6:**
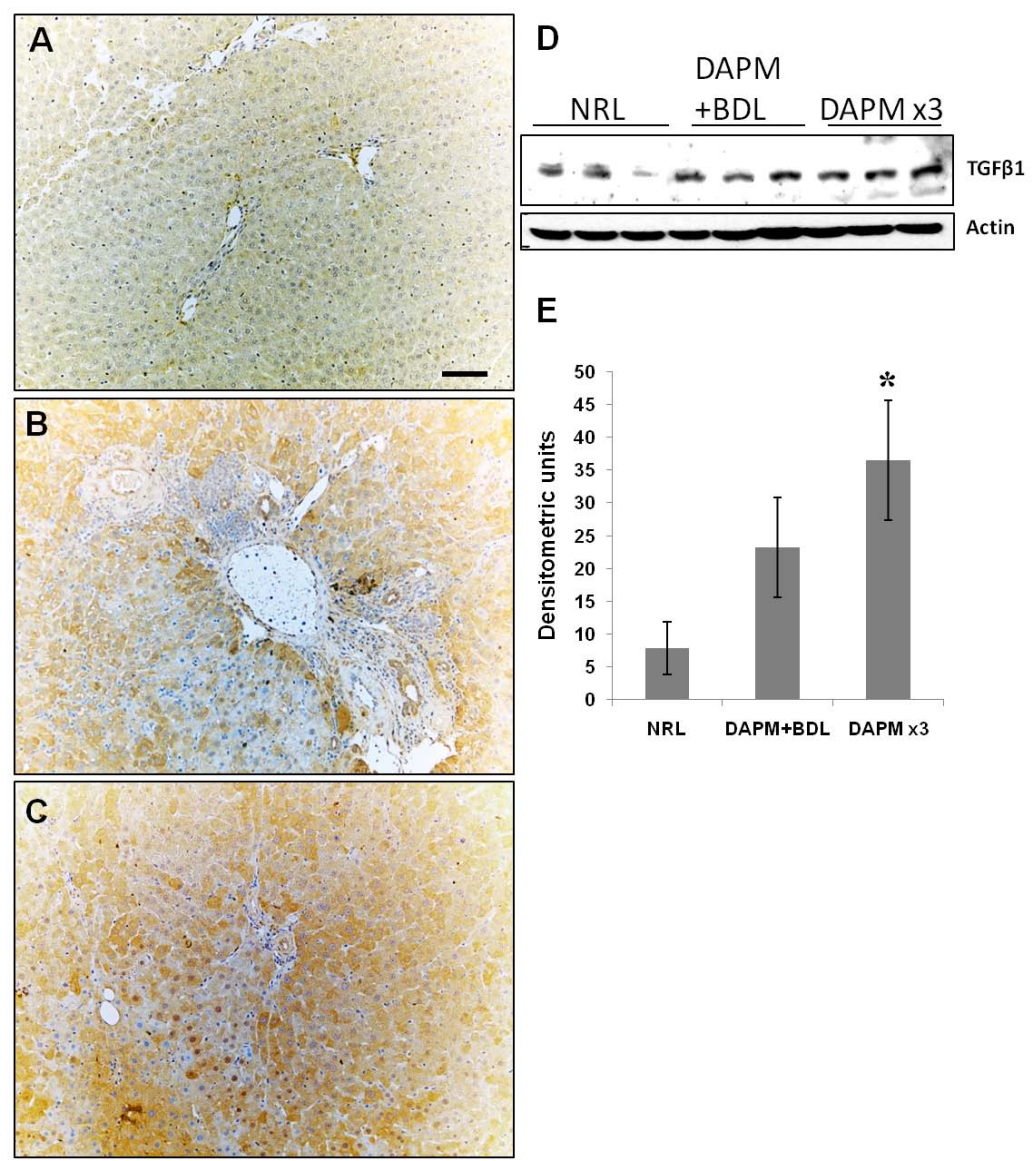
**TGFβ1 immunohistochemistry**. Induction of TGFβ1 in the periportal region after DAPM + BDL **(B) **and DAPM × 3 treatment **(C) **was observed compared to NRL **(A)**. Western blot analysis of TGFβ1 after DAPM + BDL and DAPM × 3 treatment using liver whole cell lysates. *P ≤ 0.05. Scale bar = 100 μm.

Compared to controls (Figure 6A), TGFβ1 induction was observed in the region surrounding the biliary ductules after DAPM treatment in both the models under study (Figure 6B and 6C). TGFβ1 Western blot data indicated increasing trend in both the treatment protocols compared to the controls (Figure 6D), although DAPM + BDL treatment did not show statistical significance from the normal rat liver (NRL) by densitometry. In the control liver (NRL), nuclear HNF6 staining was noticed in hepatocytes and biliary cells (Additional File 2, Figure S2, A). However, after DAPM toxicity, no significant change in HNF6expression was observed (Additional File 2, Figure S2, B and C).

## Discussion

Mature hepatocytes and BECs contribute to the normal cell turnover and respond to various types of liver injuries towards self renewal [22,23]. However, when their own capacity to proliferate is compromised, both hepatocytes and BECs can act as facultative stem cells for each other and compensate for the lost liver tissue mass [1,23,24]. Presence of the full time uncommitted stem cells in the liver has been argued historically. Studies have shown that under compromised hepatocyte proliferation, biliary cells transdifferentiate into mature hepatocytes via the "oval cell" (also known as the progenitor cell) pathway [25,26]. When biliary cells are destroyed by DAPM under compromised hepatocyte proliferation, the oval cells do not emerge indicating that biliary cells are the primary source of oval cells [27,28]. Supporting this notion, hepatocyte-associated transcription factor expression by bile duct epithelium and emerging oval cells is observed in the experimental oval cell activation induced by using 2 acetyl aminofluorene (2AAF) + partial hepatectomy (PHx) model [29] and also in cirrhotic human liver [9,26].

Previously, we demonstrated that hepatocytes can also transdifferentiate into biliary cells under compromised biliary proliferation [1-4,9]. Periportal hepatocytes can transform into BEC when the latter are destroyed by DAPM and proliferation of biliary epithelium is triggered by bile duct ligation. Under this compromised biliary proliferation, biliary ducts still appeared and newly emerging ductules carried hepatocyte marker DPPIV in the chimeric liver [1]. These findings demonstrate that hepatocytes serve as facultative stem cells for the biliary epithelium upon need. In the present study, a novel rodent model of repeated biliary injury was established by repeated low dose of DAPM given to rats. Using this novel model of repeated DAPM treatment regimen, we demonstrate that hepatocytes undergo transdifferentiation into biliary epithelium also during progressive biliary damage. DAPM produces specific injury to the biliary cells because its toxic metabolites are excreted in bile [10,11]. In the DPPIV chimeric rats, bile ducts do not express DPPIV before DAPM administration; however, after repeated DAPM treatment ~20% of the biliary ductules express DPPIV, indicating that they are derived from hepatocytes. In the chimeric liver, 50% of the hepatocytes are derived from DPPIV + donor liver.

Therefore, it is possible that DPPIV negative hepatocytes also transform into BEC, however cannot be captured due to lack of DPPIV tag. As per the assumption ~40-50% ducts are derived by transdifferentiation (~20 + % by DPPIV-positive hepatocytes + ~20 + % by DPPIV-negative hepatocytes). The rest of the ducts did not require repair because of lack of injury while part of the restoration can be due to some biliary regeneration itself that escaped repeated DAPM injury. After single DAPM injection, ~70% of the ducts were injured.

DPPIV is expressed only in the hepatocytes in the chimeric rats before DAPM treatment and therefore provides strong evidence that DPPIV-positive biliary cells are originated from hepatocytes after DAPM treatment. The longest time point studied in the present study is 30 days after the DAPM treatment when biliary restoration is still underway. It is possible that the biliary cells derived from hepatocytes will suspend the expression of DPPIV as the restoration process come to an end.

It can be argued that the biliary cells from the donor liver are the source of new biliary cells observed in the chimeric liver. However, after collagenase perfusion of the donor liver only <5% contamination of small admixture of nonparenchymal cells including biliary, stellate, endothelial, and other cell types was noticed as in routine hepatocyte preparations. In addition, the chimeric rats are treated with DAPM that targets biliary cells specifically. Therefore it is unlikely that newly appearing biliary cells originate from the very small if any biliary contamination engrafted in the chimeric liver. In the chimeric rats, after a thorough examination, not a single DPPIV-positive bile duct epithelial cell was observed in total 45 portal triads examined in the sections taken randomly. DPPIV positive biliary cells are observed in the chimeric liver only after the DAPM treatment regimen.

During liver development both hepatocytes and BECs differentiate from hepatoblasts. The lineage-specific differentiation is regulated by cell-specific gene expression in turn controlled primarily by distinct sets of transcription factors [30,31]. Altered patterns of cell specificity in the expression of the transcription factors between hepatocytes and BECs has been observed under severe hepatic necrosis and chronic biliary disease in human patients [9,26] as well as in experimental conditions of 2AAF + PHx treatment [29]. In the present study, expression of the hepatocyte-specific transcription factor HNF4α was observed in the newly repairing ductules after DAPM + BDL and repeated DAPM injury. The newly repaired biliary ductules showed appearance of hepatocyte-like cells carrying HNF4α expression. It is interesting to note that the level of the HNF4α expression in repairing ductular cells was lower compared to normal hepatocytes suggesting its gradual loss during reprogramming towards biliary phenotype.

Consistent with that notion, HNF4α expressing ductular cells also expressed HNF1β, a BEC-specific transcription factor. Specific inactivation of Hnf1β gene in hepatocytes and bile duct cells using the Cre/loxP system results in abnormalities of the gallbladder and intrahepatic bile ducts, suggesting an essential function of Hnf1β in bile duct morphogenesis [17]. Gain of expression of HNF1β by the hepatocytes normally expressing HNF4α indicates switch to the biliary specification of these cells.

In order to examine if the mechanisms that govern the differentiation of hepatoblasts into BECs are recapitulated during transdifferentiation of mature hepatocytes into BECs, expression of TGFβ1 and Onecut factor HNF6 were assessed. During liver embryogenesis, a gradient of TGFβ signaling has been shown to control ductal plate hepatoblasts differentiation [20]. High TGFβ1 signaling is observed near the portal vein and is considered responsible for differentiation of hepatoblasts into biliary cells. The Onecut transcription factor HNF6, not expressed in the immediate periportal hepatoblasts inhibits TGFβ signaling in the parenchyma, and this allows normal hepatocyte differentiation. In the present study, an induction of TGFβ1 was observed in the hepatocytes the area surrounding the repairing biliary ductules, reminiscent of the changes seen in embryonic development. However, HNF6 immunohistochemistry did not reveal significant changes after DAPM treatment in both the models under study. TGFβ1 induction was also observed in the *in vitro *hepatocyte organoid cultures undergoing biliary transdifferentiation [4]. Recently, TGFβ1-treated fetal hepatocytes were found to behave as liver progenitors and also gain expression of CK19 [24]. The data from our study suggest that TGFβ1 signaling can lead to transdifferentiation without any changes in the HNF6 expression in the adult liver upon need. It is possible that other transcription factors like OC-2 known to have overlapping target genes of HNF6 [32] may be responsible for the TGFβ1 increase in the periportal hepatocytes.

The periportal hepatocytes expressed CK19 after DAPM challenge with or without BDL pointing to the source of the likely pool of hepatocytes capable of undergoing transdifferentiation. These results are also consistent with our previous findings indicating that subpopulation of periportal hepatocytes represents the progenitor pool from which biliary cells may emerge in situations of compromised biliary proliferation [1].

Taken together the findings from this study indicate that the hepatocytes constitute facultative stem cells for the biliary cells capable of repairing liver histology when the classic biliary regeneration fails. The findings also suggest that subpopulations of hepatocytes in periportal region may have a higher tendency to function as facultative stem cells compared to other cells of their kind, even though they function as hepatocytes under normal circumstances. The exact molecular mechanisms that govern interchange in expression of cell-specific HNFs remain to be elucidated. Our earlier study with hepatocyte organoid cultures point to the role of HGF and EGF in hepatobiliary transdifferentiation [4]. Via AKT independent PI3 kinase pathway, HGF and EGF promote hepatocyte to BEC transdifferentiation [4]. It is also known that Foxo transcription factors are regulated by the PI3 kinase/AKT pathway [33]. It is possible that similar signaling occurs through HGF and/or EGF via PI3 kinase regulating expression of HNF transcription factors that in turn lead to transdifferentiation. Overall, understanding of transdifferentiation of native hepatocytes and BECs may prove to be pivotal in cellular therapy against liver diseases.

## Conclusions

Under compromised biliary regeneration, transdifferentiation of hepatocytes into biliary cells provides a rescue mechanism. Periportal hepatocytes undergoing transdifferentiation gradually loose the expression of hepatocyte master regulator HNF4α and acquire HNF1β that shifts cellular profile towards biliary lineage. An increase in TGFβ1 expression in periportal region also appears to be important for the shift from hepatocytic to biliary cellular profile.

## Methods

### Materials

Collagenase for hepatocyte isolation was obtained from Boehringer Mannheim (Mannheim, Germany). General reagents and 4,4'-Methylenedianiline (DAPM) were obtained from Sigma Chemical Co. (St. Louis, MO). Primary antibodies used are: CK19 (Dako Corp; 1:100), HNF4α (Santa Cruz; 1:50), HNF6 (Santa Cruz; 1:50), HNF1β (Santa Cruz; 1:100), TGFβ1 (Santa Cruz; 1:200). Biotinylated secondary antibodies were obtained from Jackson Laboratories. Target retrieval solution was obtained from Dako Corp. ABC kit and diaminobenzidine (DAB) kit were from Vector Laboratories.

### Animals

DPPIV positive Fisher 344 male rats were obtained from Charles River Laboratories (Frederick, MD). DPPIV negative Fisher 344 male rats were obtained from Harlan (Indianapolis, IN). The animal husbandry and all procedures performed on the rats employed for these studies were approved under the IACUC protocol #0507596B-2 and conducted according to National Institute of Health guidelines.

### Generation of rats with chimeric livers

DPPIV chimeric livers were generated as previously described [3,21]. Briefly, male DPPIV negative Fisher rats (200 g) were given two intraperitoneal injections of retrorsine (30 mg/kg), dissolved in water. The injections were given 15 days apart. A month after the last injection, the rats were subjected to PHx. During the PHx operation, the rats were also injected directly into the portal circulation (via a peripheral branch of the superior mesenteric vein) with 3.5 million hepatocytes isolated from DPPIV positive male Fisher rats (200 g). The animals were left to recover and were not subjected to any other experimental procedures for the next 3 months. Assessment of the degree of engraftment was made under direct microscopic observation of sections from the chimeric livers, stained for DPPIV. The percentage of DPPIV positive and negative cells was estimated at 40× magnification in optic fields that included at least one portal triad and one central vein. The percentage of DPPIV-positive cells varied from one lobule to another. The range of engraftment per optic field (as defined above) within each animal varied from 30% to 60%.

### Treatment with DAPM

Biliary toxicant DAPM (50 mg/kg, dissolved in DMSO at a concentration of 50 mg/ml) was injected intraperitoneally to either DPPIV chimeric or DPPIV positive male Fisher 344 rats every 2 days. In the pilot study, bile duct injury after single injection of DAPM was at its peak at 24 and 48 h after treatment (Figure 1A, B) while PCNA analysis indicated that the biliary cells begin cell division at 48 h (Figure 1C). Based on these findings, we chose to administer DAPM (50 mg/kg, ip) every 2 days. This treatment was continued for total 3 times and the rats were sacrificed at day 30 after the last DAPM injection (Figure 2A). The livers were harvested and utilized for DPPIV histochemistry.

Additional two groups of normal rats ware given either intraperitoneal injection of 50 mg DAPM/kg every two days for 3 times (DAPM × 3) or single DAPM injection (50 mg DAPM/kg) two days before the bile duct ligation (DAPM+BDL). At the end of 30 days after the last treatment, rats were sacrificed Blood was collected for serum analysis. Livers were harvested for further analysis.

### Bile duct ligation

Bile duct ligation was performed as previously described [3]. Briefly, the animals were subjected to a mid-abdominal incision 3 cm long, under general anesthesia. The common bile duct was ligated in two adjacent positions approximately 1 cm from the porta hepatis. The duct was then severed by incision between the two sites of ligation.

### Immunohistochemistry

Paraffin-embedded liver sections (4 μm thick) were used for immunohistochemical staining. For HNF4α and HNF6 staining, antigen retrieval was achieved by steaming the slides 60 minutes in preheated target retrieval solution (Dako Corporation). For CK19 staining the slides were steamed for 20 minutes in high pH target retrieval solution (Dako Corporation) before blocking.

For TGFβ1 staining no antigen retrieval was necessary. The tissue sections were blocked in blue blocker for 20 minutes followed by incubation with pertinent primary antibody overnight at 4°C. The primary antibody was then linked to biotinylated secondary antibody followed by routine avidin-biotin complex method. Diaminobenzidine was used as the chromogen, which resulted in a brown reaction product.

## Competing interests

The authors declare that they have no competing interests.

## Authors' contributions

PL and WB conducted the animal studies, PL and AO performed the immunohistochemical stainings, PL and UA collected tissues and performed Western blotting, PL wrote the manuscript, UA reviewed the manuscript, GM designed the study, examined histological and immunohistochemical stainings, and reviewed the manuscript. All the authors have read and approved the final manuscript.

## Supplementary Material

Additional file 1 **Serum ALT levels in F344 rats**. Serum ALT levels after DAPM (50 mg/kg) administration in F344 rats over a time course, where * indicates statistical difference from the 0h control (P ≤ 0.05). Click here for file

Additional file 2 **HNF6 immunohistochemistry on liver sections**. (**A**) normal control rats (NRL, normal rat liver), (**B**) rats that underwent DAPM + BDL treatment, or (**C**) repeated DAPM treatment (DAPM × 3). Brown nuclear staining indicates HNF6 positive staining. No appreciable variation in HNF6 expression was noticed in the treatment versus control groups. Scale bar = 100 μm. Click here for file

## References

[B1] Michalopoulos GK Bowen WC Mule K Stolz DB Histological organization in hepatocyte organoid cultures Am J Pathol 2001 159 1877 1887 11696448 10.1016/S0002-9440(10)63034-9 PMC1867077

[B2] Michalopoulos GK Bowen WC Mulè K Lopez-Talavera JC Mars W Hepatocytes undergo phenotypic transformation to biliary epithelium in organoid cultures Hepatology 2002 36 278 283 10.1053/jhep.2002.34858 12143035 PMC1769334

[B3] Michalopoulos GK Barua L Bowen WC Transdifferentiation of rat hepatocytes into biliary cells after bile duct ligation and toxic biliary injury Hepatology 2005 41 535 544 10.1002/hep.20600 15726663 PMC1821079

[B4] Limaye PB Bowen WC Orr AV Luo J Tseng GC Michalopoulos GK Mechanisms of hepatocyte growth factor-mediated and epidermal growth factor-mediated signaling in transdifferentiation of rat hepatocytes to biliary epithelium Hepatology 2008 47 1702 1713 10.1002/hep.22221 18398918 PMC2615562

[B5] Nishikawa Y Doi Y Watanabe H Tokairin T Omori Y Su M Yoshioka T Enomoto K Transdifferentiation of mature rat hepatocytes into bile duct-like cells in vitro Am J Pathol 2005 166 1077 1088 15793288 10.1016/S0002-9440(10)62328-0 PMC1602375

[B6] Watanabe H Hata M Terada N Ueda H Yamada N Yamanegi K Ohyama H Kakihana M Okamura H Nakasho K Transdifferentiation into biliary ductular cells of hepatocytes transplanted into the spleen Pathology 2008 40 272 276 10.1080/00313020801911546 18428047

[B7] Desmet V Roskams T Van Eyken P Ductular reaction in the liver Pathol Res Pract 1995 191 513 524 7479372 10.1016/s0344-0338(11)80870-8

[B8] Chen YK Zhao XX Li JG Lang S Wang YM Ductular proliferation in liver tissues with severe chronic hepatitis B: an immunohistochemical study World J Gastroenterology 2006 12 1443 1446 10.3748/wjg.v12.i9.1443 PMC4124327 16552818

[B9] Limaye PB Alarcón G Walls AL Nalesnik MA Michalopoulos GK Demetris AJ Ochoa ER Expression of specific hepatocyte and cholangiocyte transcription factors in human liver disease and embryonic development Lab Invest 2008 88 865 872 10.1038/labinvest.2008.56 18574450 PMC2631390

[B10] Kanz MF Gunasena GH Kaphalia L Hammond DK Syed YA A minimally toxic dose of methylene dianiline injures biliary epithelial cells in rats Toxicol Appl Pharmacol 1998 150 414 426 10.1006/taap.1998.8382 9653073

[B11] Kanz MF Wang A Campbell GA Infusion of bile from methylene dianiline-treated rats into the common bile duct injures biliary epithelial cells of recipient rats Toxicol Lett 1995 78 165 171 10.1016/0378-4274(95)03251-F 7618181

[B12] Duncan SA Transcriptional regulation of liver development Dev Dyn 2000 219 131 142 10.1002/1097-0177(2000)9999:9999<::AID-DVDY1051>3.3.CO;2-E 11002334

[B13] Zaret KS Grompe M Generation and regeneration of cells of the liver and pancreas Science 2008 322 1490 1494 10.1126/science.1161431 19056973 PMC2641009

[B14] Pontoglio M Barra J Hadchouel M Doyen A Kress C Bach JP Babinet C Yaniv M Hepatocyte nuclear factor 1 inactivation results in hepatic dysfunction, phenylketonuria, and renal Fanconi syndrome Cell 1996 84 575 585 10.1016/S0092-8674(00)81033-8 8598044

[B15] Li J Ning G Duncan SA Mammalian hepatocyte differentiation requires the transcription factor HNF-4alpha Genes Dev 2000 14 464 474 10691738 PMC316377

[B16] Hayhurst GP Strick-Marchand H Mulet C Richard AF Morosan S Kremsdorf D Weiss MC Morphogenetic competence of HNF4 alpha-deficient mouse hepatic cells J Hepatol 2008 49 384 395 10.1016/j.jhep.2008.04.024 18617288 PMC2625285

[B17] Coffinier C Gresh L Fiette L Tronche F Schütz G Babinet C Pontoglio M Yaniv M Barra J Bile system morphogenesis defects and liver dysfunction upon targeted deletion of HNF1beta Development 2002 129 1829 1838 11934849 10.1242/dev.129.8.1829

[B18] Clotman F Lannoy VJ Reber M Cereghini S Cassiman D Jacquemin P Roskams T Rousseau GG Lemaigre FP The onecut transcription factor HNF6 is required for normal development of the biliary tract Development 2002 129 1819 1828 11934848 10.1242/dev.129.8.1819

[B19] Clotman F Lemaigre FP Control of hepatic differentiation by activin/TGFbeta signaling Cell Cycle 2006 5 168 171 10.4161/cc.5.2.2341 16357531

[B20] Clotman F Jacquemin P Plumb-Rudewiez N Pierreux CE Van der Smissen P Dietz HC Courtoy PJ Rousseau GG Lemaigre FP Control of liver cell fate decision by a gradient of TGF beta signaling modulated by Onecut transcription factors Genes Dev 2005 19 1849 1854 10.1101/gad.340305 16103213 PMC1186184

[B21] Laconi E Oren R Mukhopadhyay DK Hurston E Laconi S Pani P Dabeva MD Shafritz DA Long-term, near-total liver replacement by transplantation of isolated hepatocytes in rats treated with retrorsine Am J Pathol 1998 153 319 329 9665494 10.1016/S0002-9440(10)65574-5 PMC1852941

[B22] Michalopoulos GK DeFrances MC Liver regeneration Science 1997 276 60 66 10.1126/science.276.5309.60 9082986

[B23] Michalopoulos GK Liver regeneration J Cell Physiol 2007 213 286 300 10.1002/jcp.21172 17559071 PMC2701258

[B24] del Castillo G Alvarez-Barrientos A Carmona-Cuenca I Fernández M Sánchez A Fabregat I Isolation and characterization of a putative liver progenitor population after treatment of fetal rat hepatocytes with TGF-beta J Cell Physiol 2008 215 846 855 10.1002/jcp.21370 18286537

[B25] Bisgaard HC Nagy P Santoni-Rugiu E Thorgeirsson SS Proliferation, apoptosis, and induction of hepatic transcription factors are characteristics of the early response of biliary epithelial (oval) cells to chemical carcinogens Hepatology 1996 1 62 70 10.1002/hep.510230110 8550050

[B26] Zhou H Rogler LE Teperman L Morgan G Rogler CE Identification of hepatocytic and bile ductular cell lineages and candidate stem cells in bipolar ductular reactions in cirrhotic human liver Hepatology 2007 45 716 724 10.1002/hep.21557 17326146

[B27] Petersen BE Zajac VF Michalopoulos GK Bile ductular damage induced by methylene dianiline inhibits oval cell activation Am J Pathol 1997 151 905 909 9327722 PMC1858051

[B28] Best DH Coleman WB Bile duct destruction by 4,4'-diaminodiphenylmethane does not block the small hepatocyte-like progenitor cell response in retrorsine-exposed rats Hepatology 2007 46 1611 1619 10.1002/hep.21876 17705295

[B29] Paku S Nagy P Kopper L Thorgeirsson SS 2-acetylaminofluorene dose-dependent differentiation of rat oval cells into hepatocytes: confocal and electron microscopic studies Hepatology 2004 39 1353 1361 10.1002/hep.20178 15122764

[B30] Cereghini S Liver-enriched transcription factors and hepatocyte differentiation FASEB J 1996 10 267 282 8641560

[B31] Darlington GJ Molecular mechanisms of liver development and differentiation Curr Opin Cell Biol 1999 11 678 682 10.1016/S0955-0674(99)00035-6 10600708

[B32] Jacquemin P Lannoy VJ Rousseau GG Lemaigre FP OC-2, a novel mammalian member of the ONECUT class of homeodomain transcription factors whose function in liver partially overlaps with that of hepatocyte nuclear factor-6 J Biol Chem 1999 274 2665 2671 10.1074/jbc.274.5.2665 9915796

[B33] Adachi M Osawa Y Uchinami H Kitamura T Accili D Brenner DA The forkhead transcription factor FoxO1 regulates proliferation and transdifferentiation of hepatic stellate cells Gastroenterology 2007 132 1434 1446 10.1053/j.gastro.2007.01.033 17408630

