# Supplementary figures and images for: Expression of hepatocytic- and biliary-specific transcription factors in regenerating bile ducts during hepatocyte-to-biliary epithelial cell transdifferentiation
Source: Comp Hepatol. 2010 Dec 2;9:9. doi: 10.1186/1476-5926-9-9 (PMC3014870; doi:10.1186/1476-5926-9-9)

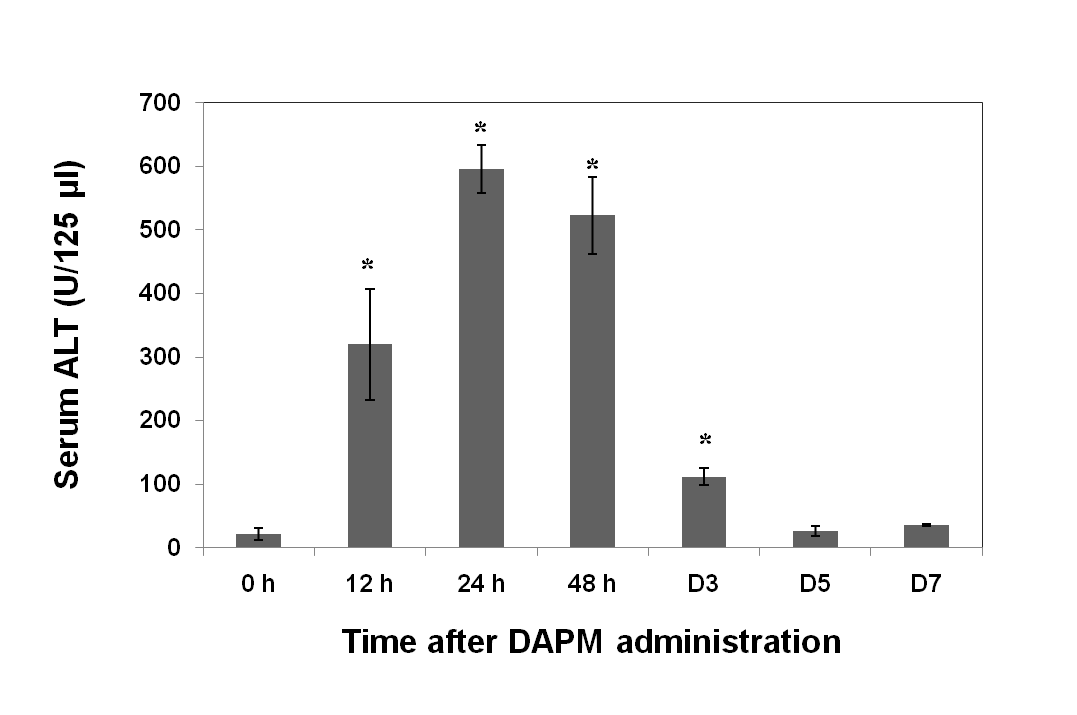

Supplement: Additional file 1 — Serum ALT levels in F344 rats. Serum ALT levels after DAPM (50 mg/kg) administration in F344 rats over a time course, where * indicates statistical difference from the 0h control (P ≤ 0.05). [file 1476-5926-9-9-S1.TIFF]

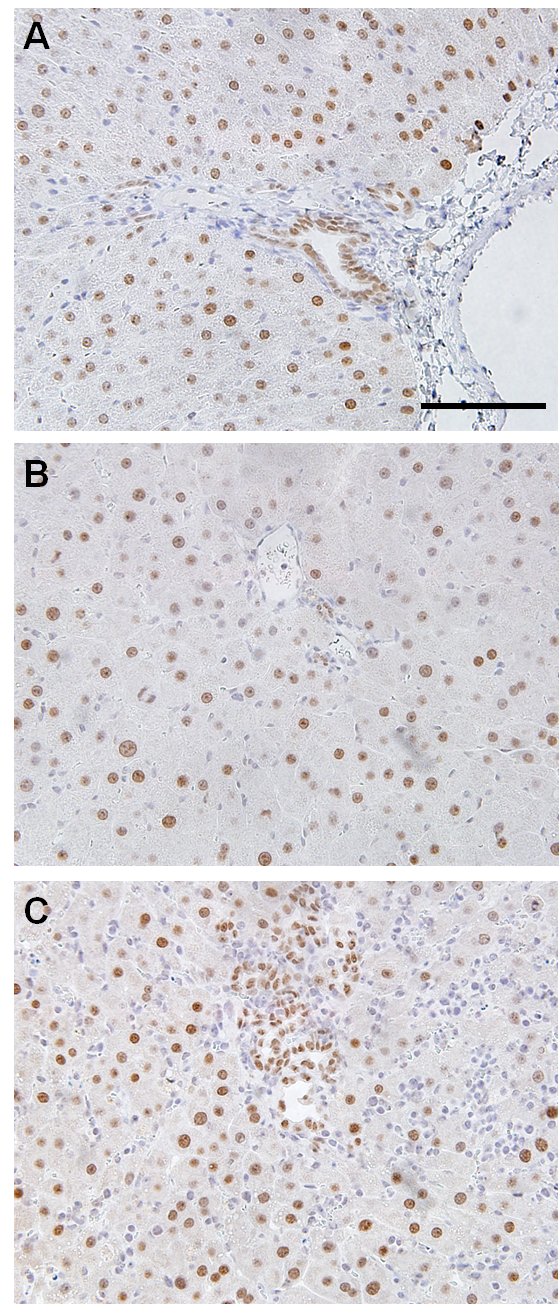

Supplement: Additional file 2 — HNF6 immunohistochemistry on liver sections. (A) normal control rats (NRL, normal rat liver), (B) rats that underwent DAPM + BDL treatment, or (C) repeated DAPM treatment (DAPM × 3). Brown nuclear staining indicates HNF6 positive staining. No appreciable variation in HNF6 expression was noticed in the treatment versus control groups. Scale bar = 100 μm. [file 1476-5926-9-9-S2.TIFF]
